# Impact of pulmonary vein isolation on atrial arrhythmias in patients with typical atrial flutter: systematic review and meta-analysis of randomized clinical trials

**DOI:** 10.1093/ehjopen/oeae102

**Published:** 2024-12-12

**Authors:** Daniel A Gomes, Rita Reis Santos, Jorge Ferreira, Frédéric Anselme, Peter Calvert, Amand Floriaan Schmidt, Dhiraj Gupta, Serge Boveda, Pedro Adragão, Rui Providência

**Affiliations:** Department of Cardiology, Hospital de Santa Cruz, Unidade Local de Saúde de Lisboa Ocidental, 2790-134 Carnaxide, Portugal; Department of Cardiology, Hospital de Santa Cruz, Unidade Local de Saúde de Lisboa Ocidental, 2790-134 Carnaxide, Portugal; Department of Cardiology, Hospital de Santa Cruz, Unidade Local de Saúde de Lisboa Ocidental, 2790-134 Carnaxide, Portugal; Service de Cardiologie CHU de Rouen, 76000 Rouen, France; Liverpool Heart & Chest Hospital NHS Foundation Trust, Liverpool L14 3PE, UK; Liverpool Centre for Cardiovascular Science, University of Liverpool, Liverpool L69 7TX, UK; Institute of Cardiovascular Science, University College London, London WC1E 6DD, UK; Department of Cardiology, Amsterdam Cardiovascular Sciences, Amsterdam University Medical Centres, University of Amsterdam, 1105 AZ Amsterdam, The Netherlands; Liverpool Heart & Chest Hospital NHS Foundation Trust, Liverpool L14 3PE, UK; Liverpool Centre for Cardiovascular Science, University of Liverpool, Liverpool L69 7TX, UK; Clinique Pasteur, Heart Rhythm Management Department, 31300 Toulouse, France; Heart Rhythm Management Centre, Universitair Ziekenhuis Brussel-Vrije Universiteit Brussel, 1090 Jette, Belgium; Department of Cardiology, Hospital de Santa Cruz, Unidade Local de Saúde de Lisboa Ocidental, 2790-134 Carnaxide, Portugal; Department of Cardiology, Rhythm Heart Centre, Hospital da Luz, 1500-650 Lisbon, Portugal; Institute of Health Informatics Research, University College London, 222 Euston Road, London NW1 2DA, UK; Barts Heart Centre, St Bartholomew’s Hospital, Barts Health NHS Trust, London EC1A 7BE, UK

**Keywords:** Atrial fibrillation, Catheter ablation, Cavotricuspid isthmus ablation

## Abstract

**Aims:**

Cavotricuspid isthmus (CTI) ablation is the current ablation treatment for typical atrial flutter (AFL). However, post-ablation atrial tachyarrhythmias, mostly in the form of atrial fibrillation (AF), are frequently observed after CTI ablation. We aimed to evaluate the effectiveness and safety of concomitant or isolated pulmonary vein isolation (PVI) in patients with typical AFL scheduled for ablation.

**Methods and results:**

Electronic databases (PubMED, EMBASE, Clinicaltrials.gov) were searched through July, 2024. Randomized controlled trials (RCTs) were eligible if comparing PVI ± CTI ablation vs. CTI alone. The primary outcomes were any sustained atrial arrhythmia, typical AFL relapse, and AF. Secondary outcomes were need for redo-ablation or antiarrhythmic drugs. Random-effects and fixed-effects meta-analyses were undertaken for each individual outcome. Seven RCTs, with a total of 902 patients, were included. Comparing to CTI ablation alone, PVI ± CTI was more effective in preventing atrial tachyarrhythmias [risk ratio (RR) = 0.57, 95% CI: 0.41–0.79, *P* = 0.0007, *I*^2^ = 50%, number needed to treat (NNT) = 4.1]. The results were driven mainly by a reduction of new onset/recurrent AF (RR = 0.41, 95% CI: 0.27–0.61, *P* < 0.0001, *I*^2^ = 0%, NNT = 3.3), whereas there were no differences in typical AFL relapse (RR = 1.52, 95% CI: 0.63–3.66, *P* = 0.35, *I*^2^ = 9%). Major complication rate was low and comparable across groups, although uncomplicated pericardial effusion was higher in PVI ± CTI (1.8% vs. 0.0%, *P* = 0.04). Results were comparable for the sub-analysis of PVI alone vs. CTI ablation.

**Conclusion:**

In patients with typical AFL, PVI ± CTI ablation is more effective than CTI alone in reducing the atrial tachyarrhythmias and subsequent AF during follow-up, without affecting major complications rate. These results set the rationale for a well-designed, larger-scale RCT comparing both strategies.

## Graphical abstract

**Graphical abstract:**
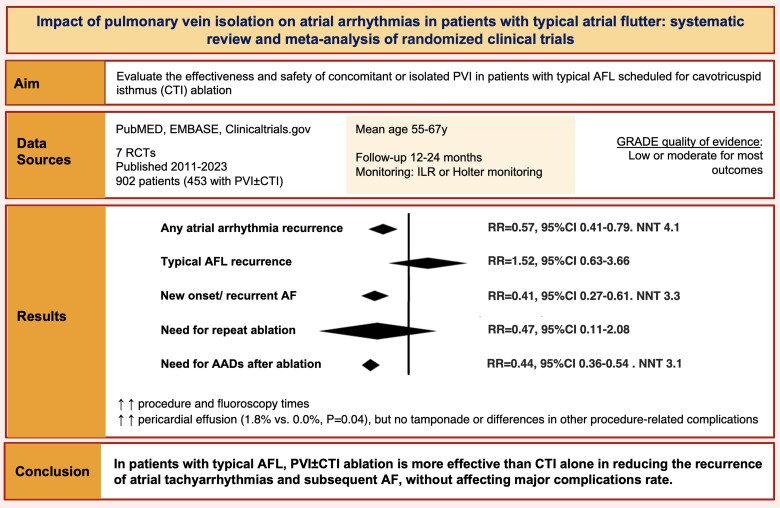

## Introduction

Atrial flutter (AFL) is an organized macro-reentrant tachycardia originating within the right atrium, most commonly in a counterclockwise fashion involving the cavotricuspid isthmus (CTI).^1^ In such cases (i.e. typical AFL), radiofrequency (RF) ablation, with achievement of bidirectional block of the CTI has a class I indication and accounts for a significant proportion of all ablation procedures worldwide.^2,3^ Despite the acute success rate being as high as 95%, more than half of the patients will develop recurrence of atrial arrhythmias during long-term follow-up, most commonly in the form of atrial fibrillation (AF).^4–7^ In fact, AFL and AF are thought to share common pathophysiological processes, as both may be triggered by pulmonary vein (PV) ectopy.^7,8^ Furthermore, they share associated comorbidities such as hypertension, heart failure, and pulmonary disease.^6,9^

Since its first description by Haïssaguerre *et al.*^10^ in 1998, pulmonary vein isolation (PVI) is increasingly recognized as mainstay of treatment for symptomatic AF, with reported long-term success rates of up to 70%.^11,12^ Over the past decade, randomized controlled trials (RCTs) have suggested that prophylactic PVI combined with CTI ablation could reduce the future development of atrial arrhythmias in patients with typical AFL without prior documentation of AF.^13,14^ More recently, an RCT of patients with typical AFL without prior documentation of AF has reported similar efficacy of PVI alone compared with CTI ablation for prevention of atrial arrhythmia recurrence.^15^ Despite these results, these studies had limitations, such as small sample size, and there is uncertainty regarding the utilized alternative strategies for AFL treatment.

We aimed to evaluate the efficacy and safety of PVI (with or without concomitant CTI ablation) in patients with typical AFL scheduled for ablation.

## Methods

This systematic review was conducted in accordance with the Preferred Reported Items for Systematic Reviews and Meta-analyses guidelines.^16^ The protocol was registered in the international prospective register of systematic reviews (PROSPERO CRD42024567879).

### Search strategy and selection criteria

We performed a systematic search of 3 electronic databases (PubMED, EMBASE, and Clinicaltrials.gov) from 1998 to 12 July 2024, using the following search string (‘atrial flutter’ AND ‘pulmonary vein isolation’). Reference lists of eligible studies were searched for additional sources of information. No language restrictions were applied.

Studies were considered eligible if they (1) were RCTs including patients aged ≥18 years with typical CTI-dependent AFL and (2) compared PVI (with or without additional ablation lines) vs. CTI ablation alone, regardless of the energy used. Two investigators (D.A.G. and R.R.S.) independently screened and selected potentially eligible studies based on title and abstract. Final eligibility was decided after evaluation of full-text publications. All disagreements were resolved via discussion or through the involvement of a third referee (R.P.).

### Data extraction and quality assessment

Data extraction was done independently by two investigators (D.A.G. and R.R.S.), and all disagreements were resolved via discussion, or through the involvement of a third referee (R.P.). A standardized form was used to extract the following information from each study: (i) study design and methodology; (ii) baseline characteristics of the participants [age, sex, medication with antiarrhythmic drugs (AADs), left atrial (LA) diameter, and left ventricular ejection fraction] and details of the ablation procedures; (iii) information on the assessment of the main clinical outcomes [ECG, Holter monitoring, implantable loop recorder (ILR)], including length of follow-up; and (iv) measures of effect and safety as stated in the protocol of the current meta-analysis.

Study authors were contacted for obtained missing data in published papers of potential interest to this review.

The risk of bias (RoB) was assessed using the Cochrane risk of bias tool.^17^ Critical assessments on the risk of bias (high, low, unclear) were done independently by two investigators (D.A.G. and R.R.S.), and all disagreements were resolved via discussion or through the involvement of a third referee (R.P.) (see Supplementary material online, *Table S1* and Supplementary material online, *Figure S1*). A trial was considered of high quality if no domains scored as high risk. Quality of evidence was appraised using GRADE criteria (see Supplementary material online, *Table S2*).

### Outcome measures

The main clinical outcomes were (i) any sustained atrial arrhythmia lasting for >30 s (AFL, atrial tachycardia, or AF) after blanking period, as assessed by 12-lead ECG, Holter monitoring, or ILR; (ii) typical CTI-dependent AFL after blanking; and (iii) post-ablation AF (after blanking). The main safety outcome was any procedure-related major complications (e.g. cardiac tamponade, major bleeding or vascular complication, peri-procedural stroke, persistent phrenic nerve palsy, atrioesophageal fistula, and death).

Additional outcomes assessed were need for repeat ablation, freedom from AADs, changes in quality of life, and procedure and fluoroscopy times.

### Data synthesis and analysis

Trial-level data were analysed according to the original randomization group. Data were synthesized if reported in at least two included studies. Continuous variables were presented as mean with standard deviation (SD) or medians and interquartile range, when appropriate. Pooled risk ratio (RR) and 95% CIs were used as summary statistics and were calculated using the DerSimonian and Laird random-effects model. Heterogeneity across studies was assessed by *I*^2^ using Cochran’s Q test, where values of <25%, 50%, and 75% were regarded as evidence of low, moderate, and high levels of heterogeneity, respectively.^18^ Funnel plots were used for evaluating the presence of publication bias for the main outcomes (see Supplementary material online, *Figure S2*).

The following planned sensitivity/sub-group analyses were done based on the following subgroups: (i) isolated PVI vs. PVI in association with CTI ablation; (ii) studies with patients with AFL and previously documented AF vs. no previously documented AF; (iii) studies with mandatory ILR follow-up vs. no mandatory ILR follow-up; and (iv) multi-centre vs. single-centre trials.

To test the consistency of the results, a sensitivity analysis for the main outcomes using a fixed-effects model was also performed.

Analyses were undertaken using Review Manager software (RevMan), V.5.4.1 (The Cochrane Collaboration, Copenhagen, Denmark).

## Results

### Study selection and patients’ characteristics

**Figure 1:**
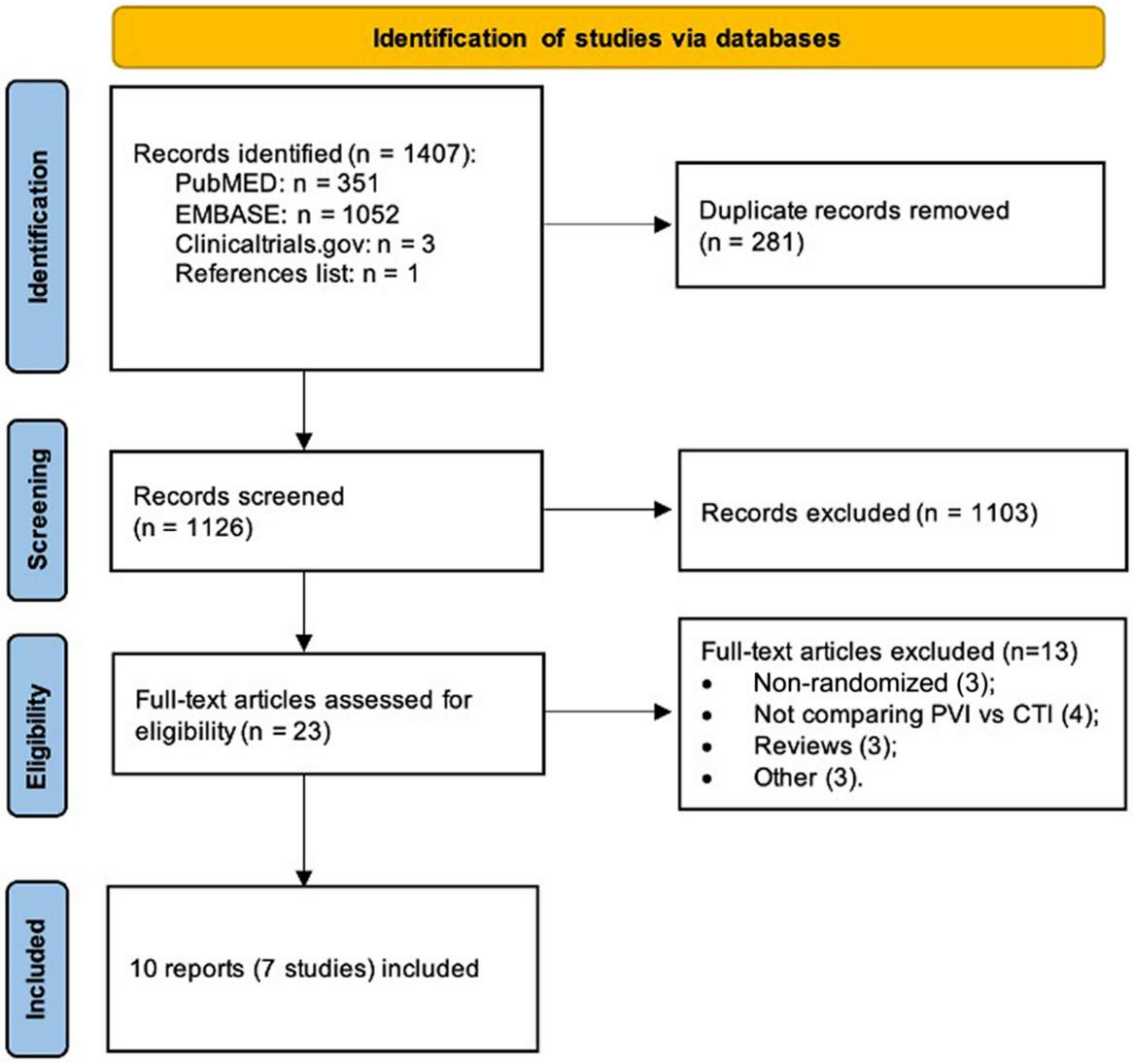
Flow diagram of the research strategy and study selection process.

A total of 1407 records were identified through database searching. Following screening, 23 reports were identified for full-text review, and further 13 were subsequently excluded (see Supplementary material online, *Table S3*). Among the excluded studies, there were two relevant systematic reviews,^19,20^ whose reference lists were checked for additional studies to add to our review (*n* = 1; *Figure 1*).

Overall, 7 different RCTs (from 10 reports) published between 2011 and 2023 with 902 patients [206 (22.8%) treated with isolated PVI and 247 (27.3%) with PVI in association with CTI ablation] were included in the final analysis.^13–15,21–27^ There was full agreement between investigators (D.G., R.R.S., and R.P.) on the inclusion of the selected studies. A summary of the studies’ characteristics is presented in *Tables 1* and *2*.

**Table 1:** Study design of the included randomized controlled trials

	Type of study	Group	Patients (*n*)	Energy	Follow-up (months)	Blanking period	Monitoring
Gupta *et al*.^15^	Multi-centre RCT, open with blind assessment	PVI	59	Cryo	12	4 weeks	ILR monitoring
		CTI	54	RF			
Anselme *et al*.^b23^	Multi-centre RCT, open label	PVI + CTI	36	Cryo + RF	24	12 weeks	12-lead ECG and 7-day Holter monitoring (weeks 12, 26, 52, 104)
		CTI	36	RF			
Mohanty *et al*.^13^	Multi-centre RCT, open label	PVI + CTI	108	RF	18 ± 6	12 weeks	ILR or 7-day Holter every 3 months
		CTI	108	RF			
Schneider *et al*.^21^	Single-centre, open-label	PVI	20	RF	17 ± 10	No blanking	ILR or 7-day Holter at 3 and 6 months, and every 6 months thereafter
		CTI	23	RF			
Steinberg *et al*.^14^	Single-centre, single-blind	PVI + CTI	25	Cryo + RF	12	12 weeks	ILR monitoring
		CTI	25	RF			
Mohanty *et al*.^b24^	Multi-centre RCT, single-blind	PVI ± CTI^a^	182	RF	21 ± 9	12 weeks	12-lead ECG and 7-day Holter monitoring (months 3, 6, 9, 12)
		CTI	178	RF			
Navarrete *et al*.^22^	Single-centre, open-label	PVI + CTI	23	RF	16 ± 4	8 weeks	Monthly visit and 48-h-Holter monitoring (every 2 months)
		CTI	25	RF			

Cryo, cryoablation; CTI, cavotricuspid isthmus; PVI, pulmonary vein isolation; RCT, randomized clinical trial; RF, radiofrequency.

^a^124 patients received PVI only; 58 patients PVI + CTI.

^b^Anselme *et al*.^23^ and Mohanty *et al*.^24^ included patients with both AFL and AF.

**Table 2:** Baseline characteristics of the included randomized controlled trials

	Group	Age (years)	Male (%)	LA diameter (mm)	LVEF (%)	AADs class I or III (%)	CHA_2_DS_2_-VASc score
Gupta *et al*.^15^	PVI	66 (61–71	88	40 (36–44	55 (49–60)	10.2	1 (0–2)
	CTI	67 (70–73)	85	42 (38–45)	55 (50–60)	11.1	1 (1–2)
Anselme *et al*.^23^	PVI + CTI	62.1 ± 8.6	83	NR	NR	77.8	NR
	CTI	65.6 ± 9.7	72			69.4	
Mohanty *et al*.^13^	PVI + CTI	62.4 ± 9.3	73	45 ± 6	57 ± 11	NR	NR
	CTI	61.2 ± 9.7	75	46 ± 8	59 ± 10		
Schneider *et al*.^21^	PVI	61.1 ± 10	75	46 ± 7	54 ± 14	NR	NR
	CTI	63.9 ± 7.9	91	43 ± 7	56 ± 12		
Steinberg *et al*.^14^	PVI + CTI	57.3 ± 9.0	76	51.9 ± 2.7	56 ± 3	NR	1.6 ± 1.0
	CTI	56.7 ± 10.0	52	51.1 ± 3.2	55 ± 4		1.96 ± 0.9
Mohanty *et al*.^24^	PVI ± CTI	61 ± 10	76	43 ± 7	59 ± 8	67	NR
	CTI	62 ± 9	76	42 ± 8	58 ± 10	75	
Navarrete *et al*.^22^	PVI + CTI	56 ± 6	NR	42 ± 1	NR	NR	NR
	CTI	55 ± 5		41 ± 1			

AAD, antiarrhythmic drug; CTI, cavotricuspid isthmus; LA, left atrium; LVEF, left ventricular ejection fraction; NR, not reported; PVI, pulmonary vein isolation; RCT, randomized clinical trial; RF, radiofrequency.

Five trials^13–15,21,22^ only included patients with isolated typical AFL, whereas in the remaining two,^23,24^ participants also had history of concomitant AF. In Schneider *et al*.^21^ and Gupta *et al*.,^15^ the study intervention was isolated PVI, and, in the 2013 RCT by Mohanty *et al*.,^24^ patients randomized to the experimental arm underwent PVI with or without concomitant CTI ablation, according to the presence of CTI-dependent AFL during catheter manipulation. In one study, six patients randomized to PVI (24%) also underwent additional ablation lines.^22^ In all cases, point-by-point RF energy was used for CTI isolation as was for PVI in the majority of the included trials.

Mean age ranged from 55 to 67 years, with men accounting for most of the included patients. Mean LA diameter varied from 40 to 52 mm, and the majority had baseline preserved left ventricular ejection fraction. The rate of utilization of AADs prior to catheter ablation differed substantially, with one trial referring patients for an early ablation strategy.^15^

According to the Cochrane Collaboration’s tool for assessing the RoB, none of the included RCTs was classified as high quality (see Supplementary material online, *Table S1* and Supplementary material online, *Figure S1*).

### Main outcomes

**Figure 2:**
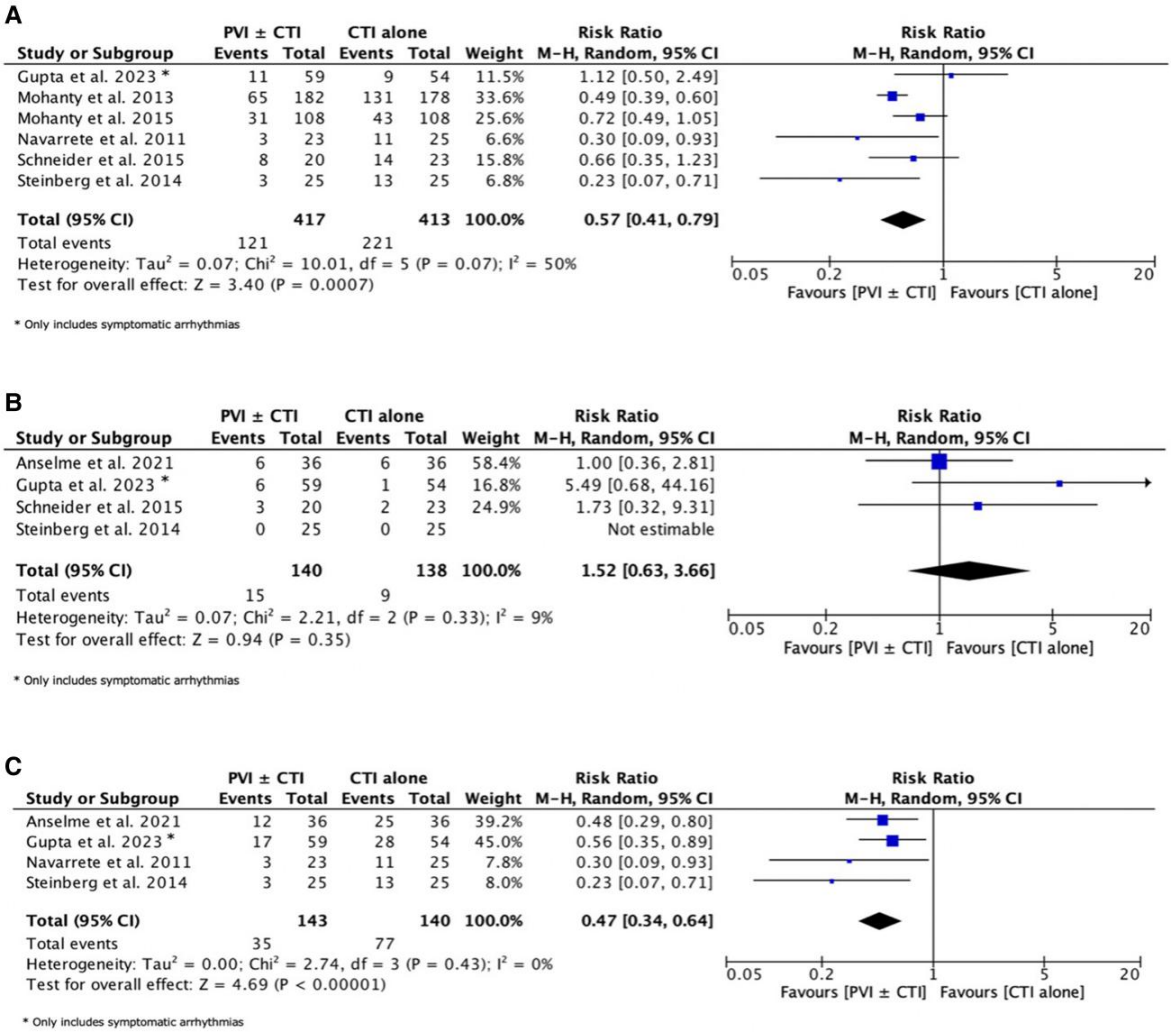
Recurrence of any atrial arrhythmia during follow-up (*A*), typical atrial flutter recurrence during follow-up (*B*), and new-onset or recurrence atrial fibrillation during follow-up (*C*).

The follow-up period ranged from 12 to 24 months, and outcome assessment was obtained through ILR or long-term Holter monitoring in most studies. Six trials reported the incidence of any sustained atrial arrhythmia during follow-up.^13–15,21,22,24^ The definition used for this outcome in each RCT is reported in Supplementary material online, *Table S4*. In Gupta *et al.*^15^, only symptomatic arrhythmias were reported. Pooling of data showed that 121 of 417 patients (29.0%) treated with PVI ± CTI ablation experienced atrial arrhythmias during follow-up vs. 221/413 (53.5%) in the control group: RR = 0.57, 95% CI: 0.41–0.79, *P* = 0.0007, *I*^2^ = 50% (*Figure 2*A). The number needed to treat (NNT) in the intervention arm to prevent one sustained atrial arrhythmia was 4.1 patients.

The recurrence of typical AFL during follow-up was reported in four trials,^14,15,21,23^ and no significant differences were observed for the compared procedures (10.7% vs. 6.5%, RR = 1.52, 95% CI: 0.63–3.66, *P* = 0.35, *I*^2^ = 9%, *Figure 2B*). On the other hand, the incidence of recurrent or new-onset AF following ablation was reduced by more than half in the intervention arm (15.4% vs. 40.7%, RR = 0.41, 95% CI: 0.27–0.61, *P* < 0.0001, *I*^2^ = 0%, NNT = 3.3, *Figure 2C*).

### Secondary outcomes

Thirty-eight patients (13.4%) underwent repeat ablation procedures during follow-up. No significant differences between groups were observed, despite a numerically higher rate in the CTI group (9.1% vs. 17.9%, RR = 0.47, 95% CI: 0.11–2.08, *P* = 0.32, *I*^2^ = 66%, see Supplementary material online, *Figure S3*). Nonetheless, the need for AADs after blanking was significantly lower in the PVI ± CTI group, mainly driven by the results of Mohanty *et al*.^24^ (RR = 0.44, 95% CI: 0.36–0.54, *P* < 0.0001, *I*^2^ = 0%, NNT = 3.1; see Supplementary material online, *Figure S4*).

Outcomes on quality of life (QoL) were only evaluated in two studies.^15,24^ In both cases, patients reported improvement in QoL after ablation, but there were no differences between groups for most of the parameters evaluated (see Supplementary material online, *Table S5*).

Logically, both procedure duration (151 vs. 72 min) and fluoroscopy time (30 vs. 10 min) were longer in the PVI ± CTI-treated group: mean difference of 86.9, 95% CI: 25.8–148.0, *P* = 0.005, *I*^2^ = 99%, and 20.0, 95% CI: 6.5–33.6, *P* = 0.004, *I*^2^ = 99%, respectively (see Supplementary material online, *Figure S5*).

### Safety outcomes

The incidence of pericardial effusion was significantly higher in the PVI ± CTI group (1.8% vs. 0.0%, *P* = 0.04, NNT = 56.6 patients), although it was managed conservatively in all cases and no cardiac tamponades were reported. Vascular complications, including haematoma and femoral pseudoaneurysm, occurred in 0.9% of patients in the intervention group and in 0.5% in controls. (*P* = 0.43). Similarly, there were no differences in the incidence of procedure-related stroke, with one case being reported in each group. There were no reports of procedure-related deaths (*Table 3*).

**Table 3:** Description of procedure-related complications

Procedure-related complications (*n*)	Gupta *et al*.^15^	Anselme *et al*.^23^	Mohanty *et al*.^13^	Schneider *et al*.^21^	Steinberg *et al*.^14^	Mohanty *et al*.^24^	Navarrete *et al*.^22^	PVI ± CTI (*n* = 433)	Isolated CTI (*n* = 426)
Stroke	0	2	0	*0*	0	0	0	1	1
Pericardial effusion (tamponade)	1 (0)	1 (0)	2 (0)	*0*	0	2 (0)	2 (0)	8 (0)	0 (0)
Persistent phrenic nerve palsy	0	0	0	*0*	0	0	0	0	0
Vascular complications	1	0	5	*NR*	0	0	0	4	2
Pacemaker implantation	3	1	0	*0*	0	0	0	0	4
Atrioesophageal fistula	0	0	0	*0*	0	0	0	0	0
Pulmonary vein stenosis	0	1	0	*0*	0	0	0	1	0

CTI, cavotricuspid isthmus; NR, not reported; PVI, pulmonary vein isolation.

### Sub-group and sensitivity analysis

In two of the included RCTs, the intervention was limited to PVI (without additional CTI ablation) in patients with typical AFL and no previous history of AF.^15,21^ For this sub-group of patients, there were no significant differences between groups when assessing the incidence of sustained atrial arrhythmias (24.1% % vs. 29.9%, RR = 0.81, 95% CI: 0.48–1.36, *P* = 0.43, *I*^2^ = 9%), typical AFL (11.4% vs. 3.9%, RR = 2.73, 95% CI: 0.74–10.11, *P* = 0.13, *I*^2^ = 0%, NNT = 13.3), or new-onset AF (6.8% vs. 14.8%, RR = 0.46, 95% CI: 0.15–1.43, *P* = 0.18) during follow-up (see Supplementary material online, *Figures S6*–*S8*).

For patients who underwent PVI combined with CTI isolation, a 50% risk reduction of atrial arrhythmias during follow-up was observed (30.2% vs. 58.9%, RR = 0.50, 95% CI: 0.35–0.72, *P* = 0.0002, *I*^2^ = 52%, NNT = 3.5), mainly explained by a reduction of new onset AF (21.4% vs. 57.0%, RR = 0.40, 95% CI: 0.26–0.62, *P* < 0.0001, *I*^2^ = 0%, NNT = 2.8; see Supplementary material online, *Figures S6*–*S8*). Comparable findings were obtained even after excluding RCTs involving patients with a previous diagnosis of AF: 23.9% vs. 38.3%, RR = 0.61, 95% CI: 0.39–0.94, *P* = 0.03, *I*^2^ = 44%, and 21.5% vs. 50.0%, RR = 0.41, 95% CI: 0.24–0.72, *P* = 0.002, *I*^2^ = 27%, respectively (*Table 4*). Results were also consistent among studies with and without mandatory ILR follow-up, as well as for multi-centre vs. single-centre trials and trials published in the last 5 years vs. >5 years ago (*Table 4*).

**Table 4:** Sub-analyses and sensitivity analyses

Scenario	Outcome (REF)	Events % vs. %	RR (95% CI)	*P*	*I* ^2^	NNT
PVI + CTI	Atrial arrhythmia recurrence^13,14,22,24^	30.2% vs. 58.9%	0.50 (0.35–0.72)	0.0002	52%	3.5
	Recurrent AFL^14,23^	9.8% vs. 9.8%	1.00 (0.36–2.81)	1.00	–	–
	New onset/recurrent AF^14,22,23^	21.4% vs. 57.0%	0.40 (0.26–0.62)	<0.0001	0%	2.8
PVI alone	Atrial arrhythmia recurrence^15,21^	24.1% vs. 29.9%	0.81 (0.48–1.36)	0.43	9%	–
	Recurrent AFL^15,21^	11.4% vs. 3.9%	2.73 (0.74–10.11)	0.13	0%	–
	New onset/recurrent AF^15^	6.8% vs. 14.8%	0.46 (0.15–1.43)	0.18	–	–
Previous AF	Atrial arrhythmia recurrence^24^	35.7% vs. 73.6%	0.49 (0.39–0.60)	<0.0001	–	2.6
	Recurrent AFL^23^	9.8% vs. 9.8%	1.00 (0.36–2.81)	1.00	–	–
	New onset/recurrent AF^23^	33.3% vs. 69.4%	0.48 (0.29–0.40)	0.005	–	2.8
No previous AF	Atrial arrhythmia recurrence^13–15,21,22^	23.9% vs. 38.3%	0.61 (0.39–0.94)	0.03	44%	6.9
	Recurrent AFL^14,15,21^	11.4% vs. 3.9%	2.73 (0.74–10.11)	0.13	0%	–
	New onset/recurrent AF^14,15,22^	21.5% vs. 50.0%	0.41 (0.24–0.72)	0.002	27%	3.5
Mandatory ILR follow-up	Atrial arrhythmia recurrence^14,15^	16.7% vs. 27.8%	0.53 (0.11–2.53)	0.43	80%	–
	Recurrent AFL^14,15^	7.1% vs. 1.3%	5.49 (0.68–44.16)	0.11	–	–
	New onset/recurrent AF^14,15^	8.3% vs. 26.7%	0.32 (0.15–0.72)	0.006	0%	5.5
No mandatory ILR follow-up	Atrial arrhythmia recurrence^13,21,22,24^	32.1% vs. 59.6%	0.56 (0.42–0.73)	<0.0001	35%	3.6
	Recurrent AFL^21,23^	16.1% vs. 13.6%	1.16 (0.48–2.80)	0.74	0%	–
	New onset/recurrent AF^22,23^	25.4% vs. 59.0%	0.44 (0.28–0.71)	0.0006	0%	3.0
Multi-centre studies	Atrial arrhythmia recurrence^13,15,24^	30.7% vs. 53.8%	0.65 (0.43–0.99)	0.04	69%	4.3
	Recurrent AFL^15,23^	12.6% vs. 7.8%	1.85 (0.35–9.71)	0.47	54%	–
	New onset/recurrent AF^22,23^	30.1% vs. 58.9%	0.52 (0.37–0.74)	0.0002	0%	3.5
Single-centre studies	Atrial arrhythmia recurrence^14,21,22^	20.6% vs. 56.1%	0.41 (0.20–0.83)	0.01	44%	3.2
	Recurrent AFL^14,21^	6.7% vs. 4.2%	1.73 (0.32–9.31)	0.53	–	–
	New onset/recurrent AF^14,22^	12.5% vs. 48.0%	0.26 (0.12–0.58)	0.001	0%	2.8
Studies published in the last 5 years	Atrial arrhythmia recurrence^15^	16.7% vs. 18.6%	1.12 (0.50–2.59)	0.78	–	–
	Recurrent AFL^15,23^	12.6% vs. 7.8%	1.85 (0.35–9.71)	0.47	54%	–
	New onset/recurrent AF^15,23^	30.5% vs. 58.9%	0.52 (0.37–0.74)	0.0002	0%	3.5
Studies published in > 5 years ago	Atrial arrhythmia recurrence^13,14,21,22,24^	30.7% vs. 59.1%	0.53 (0.39–0.71)	<0.0001	41%	3.5
	Recurrent AFL^14,21^	6.7% vs. 4.2%	1.73 (0.32–9.31)	0.53	–	–
	New onset/recurrent AF^14,21^	12.5% vs. 48.0%	0.26 (0.12–0.58)	0.001	0%	2.8

AF, atrial fibrillation; AFL, atrial flutter; CTI, cavotricuspid isthmus; ILR, implantable loop recorder; PVI, pulmonary vein isolation; RR, risk ratio; NNT, number needed to treat.

Sensitivity analyses using a fixed-effects model were consistent with the primary analysis for the main outcomes (see Supplementary material online, *Table S6*).

## Discussion

In this systematic review, we have explored the potential impact of PVI alone, or combined with CTI, in patients with typical AFL scheduled for ablation. The main findings were as follows:

- PVI with concomitant CTI ablation is more effective than isolated CTI ablation at preventing recurrent atrial tachyarrhythmia. The NNT to avoid one relapse was four patients, primarily due to a 50% reduction in the subsequent risk of AF.
- Stand-alone PVI showed no significant differences in AFL recurrence when compared with CTI ablation.
- Isolated CTI ablation is highly effective and safe in preventing AFL relapse (93% success), although one in every three patients may develop AF during follow-up.
- Procedural and fluoroscopy times were longer for PVI ± CTI ablation.
- Major complication rate and most assessed QoL parameters were comparable across groups.

### Mechanisms of atrial arrhythmias and AF following CTI ablation

Following CTI ablation for typical AFL, most patients will later develop atrial arrhythmias, with AF being the most common form.^4,5^ It has long been recognized that both AF and AFL share common pathophysiological and electroanatomic substrates, as both may be initiated by pulmonary vein ectopy.^8^ In fact, AFL often starts after an episode of AF of variable duration, which may facilitate a functional line of block between superior and inferior vena cavae, essential to the macroreentrant circuit formation.^7,8^ While previous studies have shown that CTI ablation alone is not effective in preventing new onset AF, a trial by Wazni *et al*.^28^ including 108 patients has demonstrated that, in patients with both typical AFL and AF, PVI alone may be equally effective to PVI plus CTI ablation in preventing long-term atrial tachyarrhythmias.

### Impact on main outcomes and clinical implications

Despite both PVI with or without concomitant CTI and CTI having comparable results for the prevention of typical AFL recurrence (10% vs. 7%, *P* = 0.35), concomitant or stand-alone PVI during AFL ablation has shown to significantly reduce the incidence of atrial arrhythmia relapse (29% vs. 54%, *P* = 0.0007) during a median follow-up of 12 to 24 months. This was mainly driven by a decrease in AF incidence (15% vs. 41%, *P* < 0.0001). Conversely, previous evidence indicates that prophylactic CTI isolation during AF ablation does not effectively reduce arrhythmia recurrence.^29^ These results add to the evidence that pulmonary vein triggers may be responsible for AFL initiation in a significant proportion of patients.

The rate of procedure-related complications (including cardiac tamponade, stroke, persistent phrenic nerve palsy, permanent pacemaker implantation, vascular complications, pulmonary vein stenosis, atrioesophageal fistula, and procedure-related death) was low and did not differ between groups, despite a higher incidence of non-complicated pericardial effusion in the PVI ± CTI group (1 for every 57 patients treated with this approach instead of isolated CTI). However, the low incidence of such complications prevents definitive conclusions.

Our sub-group analyses for the main outcomes showed consistent results independently of the presence of a previous diagnosis of AF. Furthermore, whereas PVI combined with CTI ablation was superior to CTI alone in preventing the development of future atrial arrhythmias, a strategy based on stand-alone PVI, assessed in two small RCTs,^15,21^ has shown to be non-inferior regarding this endpoint. It must, however, be highlighted that although a significant reduction of new onset AF was observed with the latter approach, there was a non-statistically significant increase in AFL recurrence (11% vs. 4%) that should be clarified in future trials. Despite considerable uncertainty arising from the infrequent occurrence of AFL in both treatment arms, we cannot entirely rule out that a larger sample could have potentially shown advantage in favour of CTI ablation. A hypothetical RCT with a large enough sample, and assuming a post-ablation AFL rate comparable to what we observed, might show that for every 13 patients undergoing CTI ablation instead of PVI, 1 AFL relapse would be prevented during a 12- to 24-month follow-up period. For the remaining 12 patients, the outcome would be the same (no AFL relapse).

Previous systematic reviews, incorporating data from 357 and 672 patients across RCTs conducted from 2011 to 2018, and failing to include any recent data (see Supplementary material online, *Table S7*),^19,30^ have attempted to address this issue. In both titles, no prospective registration or quality appraisal of the evidence was performed. This is the expected standard in systematic reviews in current day and age. Authors of the two previous titles concluded that prophylactic PVI combined with CTI ablation was superior to CTI ablation alone. Our systematic review robustly expands these findings, with some additional key differences that should be noted: (i) prospective protocol registration on PROSPERO; (ii) formal risk of bias and quality of evidence assessment (GRADE classification) to help ascertain the certainty of the conclusions; (iii) pre-planned subanalyses looking at different ablation strategies (e.g. PVI combined with CTI ablation and isolated PVI), adding novel evidence on the impact of isolated PVI in patients with typical AFL without previous AF documentation, a question, which had not been previously explored in a systematic review; our pooled data from two trials demonstrated the non-inferiority of this approach.^15,21^

Although evidence is building around the benefits of PVI (alone or in association with CTI ablation) in reducing the incidence of atrial arrhythmias in patients with typical AFL undergoing ablation, this strategy has not yet been widely adopted, likely due to its higher complexity, with associated 1% to 2% risk of cardiac tamponade, and longer procedure time. In our study, the procedural and fluoroscopy duration was 2 to 3 times longer in the PVI ± CTI group. Additionally, data on longer-term follow-up is still lacking. This information is crucial to determine whether the apparent benefits achieved with PVI are maintained over time, given the possibility of late development of PVI-related LA macroreentrant tachycardia. Importantly, the 3-year outcomes of the PREVENT AF I study and the CRAFT trial show that the magnitude of effect on the primary outcomes remains consistent (HR = 0.65, 95% CI: 0.43–0.99, *P* = 0.05 and HR = 0.97, 95% CI: 0.43–2.20, *P* = 0.944, respectively).^25,26^

### Predictors of atrial arrhythmias incidence following AFL ablation

Previous studies have explored the predictors of new onset AF following CTI ablation for isolated typical AFL.^4,9,13^ Older age, pre- or intraprocedural AF, LA diameter, and AF inducibility during CTI ablation were identified as risk factors for future development of AF.^13,31,32^ In fact, preprocedural AF and inducibility during AFL ablation were both associated to almost 4-fold increase in the risk of new onset/recurrent AF.^31,32^ Furthermore, the HATCH score (hypertension, age ≥75, chronic obstructive pulmonary disease, transient ischaemic attack/stroke, and congestive heart failure), a clinical score initially developed for predicting progression of paroxysmal to persistent AF, was also shown to have a moderate discriminative power for new-onset AF after CTI ablation (AUC=0.743).^9,33^ When compared with those scoring lower, patients scoring 2 or more were found to have more than twice the risk (69% vs. 27%) of developing AF during a median follow-up of 29 months.^9^ These proposed variables should be tested in a prospective large-scale RCT to help identifying subsets of AFL patients more likely to benefit from combined or stand-alone PVI at the time of ablation.

In the trials including patients with typical AFL and no history of AF, the *de novo* AF was observed in up to 50% of patients in the control group (CTI ablation alone) during a mean follow-up of 12 to 16 months.^14,15,22^ These results are in line with previous observational data and may be useful to inform decisions on long-term anticoagulation in patients at higher embolic risk after successful CTI ablation.^3^ However, further evidence on the risk/benefit and thresholds for anticoagulation is urgently needed.

### Limitations

The results of this systematic review and meta-analysis should be interpreted considering several limitations. First, although only RCTs were included, most have limited sample size, which may have influenced the power to detect differences between groups. Accordingly, it was not possible to explore the influence of possible effect modifiers such as age, sex, and use of AADs. Furthermore, none was assessed as having high quality using the RoB tool, mainly due to performance and detection bias, which may reduce the certainty of its conclusions. As some endpoints were subjective (e.g. symptomatic arrhythmias or quality of life), detection bias may affect some of the results. On the other hand, it is unlikely that knowledge of the received intervention might lead to seeking more or less additional care (i.e. performance bias). The only way to address this issue would be a well-designed and conducted trial in the future. Second, different PVI technologies are currently used, and it may be difficult to transpose these results to some centres, namely those utilizing pulsed-field ablation, which was not used by any of the included trials. However, despite spanning for over a decade and using different sources of energy (RF and cryo) and technologies, the magnitude of effect seems comparable across trials. Third, in this review, both RCT including patients with or without past history of AF were considered. To tackle this limitation, we have performed a sub-group analysis showing similar results in both contexts. The diagnosis of prior AF strongly depends on the type of screening (i.e. opportunistic vs. systematic). The definition of arrhythmia recurrence and monitoring techniques varied between studies (with only two using mandatory ILR as per protocol), which may have direct impact on the observed recurrence rates. Use of AADs during follow-up varied between trials and may have influenced both the rate and the relative proportion of AF/AFL recurrence. Finally, high heterogeneity was observed for some of the analyses. We tried to address this by performing multiple sub-analyses and sensitivity analyses, including for the individual components of the primary outcome (i.e. AFL recurrence and AF).

## Conclusions

In patients with typical AFL scheduled for ablation, PVI ± CTI ablation is more effective than CTI alone in reducing the incidence of atrial tachyarrhythmias and subsequent AF. This benefit appears to occur at the expense of longer procedure duration and fluoroscopy time, but with no apparent increase in major complications rate. These results set the rationale for a well-conducted, larger-scale RCT aiming to clarify the magnitude of effect and to identify sub-groups of patients more likely to benefit from the combined PVI + CTI or stand-alone PVI approach.

## Supplementary Material

oeae102_Supplementary_Data

## Data Availability

The data supporting the findings of this study are available within the article and its supplementary materials.

## Lead author biography

Daniel Gomes is a Cardiology resident at Hospital de Santa Cruz in Lisbon, Portugal. He completed his medical degree at the Faculty of Medicine, University of Lisbon. He is currently dedicating to Electrophysiology and Pacing.
