# Supplementary material for: Impact of pulmonary vein isolation on atrial arrhythmias in patients with typical atrial flutter: systematic review and meta-analysis of randomized clinical trials
Source: Eur Heart J Open. 2024 Dec 12;5(1):oeae102. doi: 10.1093/ehjopen/oeae102 (PMC11668177; doi:10.1093/ehjopen/oeae102)
Supplement: oeae102_Supplementary_Data [file oeae102_supplementary_data.docx]

**SUPPLEMENTARY MATERIAL**

**Supplementary table 1** – Risk of bias assessment.

| **study ID** | **Selection bias** | | | **Reporting bias** | | **Other bias** | | **Performance bias** | | **Detection bias** | | **Attrition bias** | | **General comments** |
| --- | --- | --- | --- | --- | --- | --- | --- | --- | --- | --- | --- | --- | --- | --- |
|  | **Random sequence generation** | **Allocation concealment** | **Comments** | **Selective reporting** | **Comments** | **Other sources of bias** | **Comments** | **Blinding participants and personnel** | **Comments** | **Blinding (outcome assessment)** | **Comments** | **Incomplete outcome data** | **Comments** |  |
| **Anselme 2021** | unclear | unclear | no data on allocation consealement or on the blocks utilized for randomization | low | All planned outcomes in the published protocol were present on the mai manuscript | unclear | published protocol on clinicaltrials.gov; borderline difference in age of participants across treatment arms | high | no blinding | high | no blinding | low | decription of enrollment and drop-outs | well prepared study; NCT01521988 |
| **Gupta 2023** | low | low | both described; sealed envelopes; RedCap was used; blocks of 2 or 4; | low | All planned outcomes in the published protocol were present on the main manuscript | unclear | published protocol as manuscript and on clinicaltrials.gov; terminated early due to Covid-19 and did not meet planned primary endpoint hypothesis; funded by Medtronic | high | patient were not blinded; albeit and implantable cardiac monitor was used, the primary endpoint was symptomatic arrhythmias, and QoL reporting might be impacted | low | blinded outcome assessment | low | no patients lost to follow-up | well prepared and described study; NCT03401099; funded by Medtronic but no role in study design or data analyses; terminated early due to Covid-19 |
| **Mohanty 2013** | low | unclear | central non-stratified randomization; blocks of 4; no information on allocation concealement methods | low | All planned outcomes in the published protocol were present on the mai manuscript; albeit more outcomes were reported on the paper than in the protocol, these were not among the ones we planned for our review | unclear | published protocol on clinicaltrials.gov; some difference in the utilization of CTI ablation in the PVI arm, but this is based on presence/absence of documentation of atrial flutter | low | patients and staff were blinded | high | outcome assessors were not blinded to treatment arm | low | even though number of patients lost to follow-up is not reported, utilizing the provided rates and number of patients with relapse, we can see the full denominator was used, implying negligible number of lost to follow-up | well prepared and described study; protocol available; NCT01439386. |
| **Mohanty 2015** | low | unclear | Randomization created by computer algorithm in blocks of 10 for each centre; no data on allocation consealement | unclear | Two endpoints planned on the protocol/clinicaltrials.gov were not reported - longterm use of AADs and anticoagulants | low | published protocol on clinicaltrials.gov | high | no blinding | high | no blinding | low | No patients lost to follow-up | well prepared and described study; protocol available, NCT01710150 |
| **Navarrete 2011** | unclear | unclear | no data on allocation consealement or randomization method | unclear | No published protocol, so we have no way to assess whether all planned outcomes were reported | high | no proof of trial registration, but protocol was approved by local IRB; lack of description for most of the important aspects on a trial | unclear | no information on blinding | unclear | no information on blinding | low | No patients lost to follow-up | No proof of trial registration, but IRB approved; little information for most RoB domains |
| **Schneider 2015** | unclear | unclear | no data on randomization method and allocation consealement | low | all planned outcomes were reported | low | published protocol on clinicaltrials.gov; | high | no blinding | high | no blinding | low | No patients lost to follow-up | Registered protocol on clinicaltrials.gov NCT02051621; |
| **Steinberg 2014** | unclear | low | no data on method for sequence generation; sealed envolopes were used | low | all planned outcomes were reported | unclear | published protocol on clinicaltrials.gov; funded by Medtronic and Biosense-Webster but no information on whether these had any role in study design or analyses | low | blinding of patients | high | no blinding | high | >10% patients lost to follow-up | Registered on clinicaltrials.gov NCT01563848; unclear role for industry funders |

**Supplementary table 2 –** Summary of findings and quality of evidence appraisal according to GRADE criteria.

| **Outcome** | **Effect Size**  **95%CI**  **P** | **Studies**  **Sample size** | **NNT to prevent or cause one event** | **Heterogeneity**  **Risk of Bias Assessment** | **Indirectness**  **Imprecision**  **Publication Bias** | **Interpretation**  **Quality of Evidence/GRADE** |
| --- | --- | --- | --- | --- | --- | --- |
| **Any sustained atrial arrhythmia relapse** | RR= 0.57  0.41-0.79  P=0.0007 | 6 RCTs,  830 patients | 4.1 patients  29.0% vs. 53.5% | High heterogeneity (I^2^=50%)  RoB – ↓1 level | No indirectness  No Imprecision  No Publication Bias* | Significant reduction  Low Quality  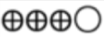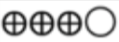 |
| **Typical atrial flutter relapse** | RR=1.52  0.63-3.66  P=0.35 | 4 RCTs  278 patients | -  10.7% vs. 6.5% | Low heterogeneity (I^2^=9%)  RoB – ↓1 level | No indirectness  Imprecision  (↓1 level)  No Publication Bias* | No differences  Low Quality  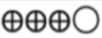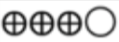 |
| **AF relapse** | RR=0.47  0.34-0.64  P<0.00001 | 4 RCTs  183 patients | 3.3 patients  15.4% vs. 40.7% | Low heterogeneity (I^2^=0%)  RoB – ↓1 level | No indirectness  No Imprecision  No Publication Bias* | Significant reduction  Moderate Quality  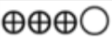 |
| **Pericardial effusion without need for intervention** | RR=4.11  1.04-16.26  P=0.04 | 7 RCTs  902 patients | 56.6 patients  1.8% vs. 0% | Low heterogeneity (I^2^=0%)  RoB – ↓1 level | No indirectness  Imprecision  (↓1 level)  No Publication Bias* | Significant increase    Low Quality  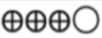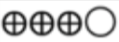 |
| **Need for repeat ablation** | RR=0.47  0.11-2.08  P=0.32 | 4 RCTs  183 patients | -  9.1% vs. 17.9% | High heterogeneity (I^2^=66%)  RoB – ↓1 level | No indirectness  Imprecision  (↓1 level)  No Publication Bias* | No differences  Very Low Quality  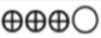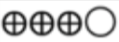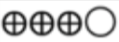 |
| **Freedom from antiarrhythmic drugs** | RR=0.44  0.36-0.54  P=0.00001 | 4 RCTs  571 patients | 3.1 patients  23.9% vs. 56.0% | Low heterogeneity (I^2^=0%)  RoB – ↓1 level | No indirectness  No Imprecision  No Publication Bias* | Significant reduction  Moderate Quality  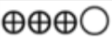 |
| **Procedure duration** | MD=86.9  25.8-148.0  P=0.005 | 4 RCTs  427 patients | - | High heterogeneity (I^2^=99%)  RoB – ↓1 level | No indirectness  Imprecision  (↓1 level)  No Publication Bias* | Significant increase  Very Low Quality  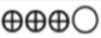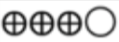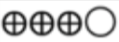 |
| **Fluroscopy duration** | MD=20.0  6.5-33.6  P=0.04 | 4 RCTs  427 patients | - | High heterogeneity (I^2^=99%)  RoB – ↓1 level | No indirectness  Imprecision  (↓1 level)  No Publication Bias* | Significant increase  Very Low Quality  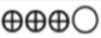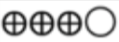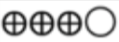 |

* Publication bias – no judgements for downgrade were performed as <10 trials were included in the systematic review

**Supplementary table 3 –** Motives for reports exclusion after full-text review.

| **Authors and year of publication** | **Title** | **Journal** | **Motive for exclusion** |
| --- | --- | --- | --- |
| Yamaji et al^1^. 2022 | Rates of atrial flutter occurrence and cavotricuspid isthmus reconduction after prophylactic isthmus ablation performed during atrial fibrillation ablation: a clinical study, review, and comparison with previous findings | J Interv Card Electrophysiol | Not comparing PVI with CTI |
| Kumar et al^2^. 2019 | Prophylactic pulmonary vein isolation during cavotricuspid isthmus ablation and period of freedom from atrial arrhythmia: A meta-analysis and TSA | Pacing Clin Electrophysiol | Letter to the editor regarding Koerber et al. 2019 |
| Peyrol et al^3^. 2015 | Simultaneous pulmonary vein cryoablation and cavotricuspid isthmus radiofrequency ablation in patients with combined atrial fibrillation and typical atrial flutter | J Electrocardiol | Not comparing PVI with CTI |
| Koerber et al^4^. 2019 | Prophylactic pulmonary vein isolation during cavotricuspid isthmus ablation for atrial flutter: A meta-analysis | Pacing Clin Electrophysiol | Systematic review |
| Fu et al^5^. 2021 | Prophylactic pulmonary vein isolation in typical atrial flutter patients without atrial fibrillation: a systematic review and meta-analysis of randomized trials | J Interv Card Electrophysiol | Systematic review |
| Dhillon et al^6^. 2014 | Feasibility and efficacy of simultaneous pulmonary vein isolation and cavotricuspid isthmus ablation using cryotherapy | J Cardiovasc Electrophysiol | Non-randomised study |
| Yoneda et al^7^. 2020 | Conduction Recovery After Cavotricuspid Isthmus Ablation When Performed With or Without Concomitant Atrial Fibrillation Ablation | JACC Clin Electrophysiol | Non-randomised study |
| Celikyurt et al^8^. 2017 | Incidence of new-onset atrial fibrillation after cavotricuspid isthmus ablation for atrial flutter | Europace | Not comparing PVI with CTI |
| Wazni et al^9^. 2003 | Randomized study comparing combined pulmonary vein-left atrial junction disconnection and cavotricuspid isthmus ablation versus pulmonary vein-left atrial junction disconnection alone in patients presenting with typical atrial flutter and atrial fibrillation | Circulation | Not comparing PVI with CTI |
| Xie et al^10^. 2018 | Prophylactic atrial fibrillation ablation in atrial flutter patients without atrial fibrillation: a meta- analysis with trial sequential analysis | Med Sci Monit Basic Res | Systematic Review |
| Gula et al^11^. 2016 | Atrial flutter and atrial fibrillation ablation – sequential or combined? A cost-benefit and risk analysis of primary prevention pulmonary vein ablation | Heart Rhythm | Cost-effectiveness study |
| Kataoka et al^12^. 2024 | How to demonstrate the advantage of cryoballoon pulmonary vein isolation over cavotricuspid isthmus ablation in patients with typical atrial flutter | J Interv Card Electrophysiol | Letter to the editor regarding the CRAFT trial |
| Aker et al^13^. 2024 | Ablation outcomes and quality of life in patients with atrial flutter and concomitant paroxysmal atrial fibrillation | Heart, Vessels and Transplantation | Non-randomised study |

**References for Supplementary table 3:**

1. Yamaji H, Higashiya S, Murakami T, Kawamura H, Murakami M, Kamikawa S, Kusachi S. Rates of atrial flutter occurrence and cavotricuspid isthmus reconduction after prophylactic isthmus ablation performed during atrial fibrillation ablation: a clinical study, review, and comparison with previous findings. J Interv Card Electrophysiol. 2022 Jun;64(1):67-76
2. Kumar A, Shariff M. Prophylactic pulmonary vein isolation during cavotricuspid isthmus ablation and period of freedom from atrial arrhythmia: A meta-analysis and TSA. Pacing Clin Electrophysiol. 2019 Aug;42(8):1163-1164
3. Peyrol M, Sbragia P, Ronchard T, Cautela J, Villacampa C, Laine M, Bonello L, Thuny F, Paganelli F, Lévy S. Simultaneous pulmonary vein cryoablation and cavotricuspid isthmus radiofrequency ablation in patients with combined atrial fibrillation and typical atrial flutter. J Electrocardiol. 2015 Jul-Aug;48(4):729-33.
4. Koerber SM, Turagam MK, Gautam S, Winterfield J, Wharton JM, Lakkireddy D, Gold MR. Prophylactic pulmonary vein isolation during cavotricuspid isthmus ablation for atrial flutter: A meta-analysis. Pacing Clin Electrophysiol. 2019 May;42(5):493-498
5. Fu B, Ran B, Zhang H, Luo Y, Wang J. Prophylactic pulmonary vein isolation in typical atrial flutter patients without atrial fibrillation: a systematic review and meta-analysis of randomized trials. J Interv Card Electrophysiol. 2021 Apr;60(3):529-533
6. Dhillon PS, Domenichini G, Gonna H, Bastiaenen R, Norman M, Gallagher MM. Feasibility and efficacy of simultaneous pulmonary vein isolation and cavotricuspid isthmus ablation using cryotherapy. J Cardiovasc Electrophysiol. 2014 Jul;25(7):714-8
7. Yoneda ZT, Shoemaker MB, Richardson T, Crawford D, Kanagasundram A, Shen S, Estrada JC, Holmes B, Lugo R, McHugh J, Saavedra P, Crossley G 3rd, Ellis CR, Montgomery JA, Michaud GF. Conduction Recovery After Cavotricuspid Isthmus Ablation When Performed With or Without Concomitant Atrial Fibrillation Ablation. JACC Clin Electrophysiol. 2020 Aug;6(8):989-996
8. Celikyurt U, Knecht S, Kuehne M, Reichlin T, Muehl A, Spies F, Osswald S, Sticherling C. Incidence of new-onset atrial fibrillation after cavotricuspid isthmus ablation for atrial flutter. Europace. 2017 Nov 1;19(11):1776-1780.
9. Wazni O, Marrouche NF, Martin DO, Gillinov AM, Saliba W, Saad E, Klein A, Bhargava M, Bash D, Schweikert R, Erciyes D, Abdul-Karim A, Brachman J, Gunther J, Pisano E, Potenza D, Fanelli R, Natale A. Randomized study comparing combined pulmonary vein-left atrial junction disconnection and cavotricuspid isthmus ablation versus pulmonary vein-left atrial junction disconnection alone in patients presenting with typical atrial flutter and atrial fibrillation. Circulation. 2003 Nov 18;108(20):2479-83
10. Xie X, Liu X, Chen B, Wang Q. Prophylactic Atrial Fibrillation Ablation in Atrial Flutter Patients without Atrial Fibrillation: A Meta-Analysis with Trial Sequential Analysis. Med Sci Monit Basic Res. 2018 Jun 30;24:96-102
11. Gula LJ, Skanes AC, Klein GJ, Jenkyn KB, Redfearn DP, Manlucu J, Roberts JD, Yee R, Tang AS, Leong-Sit P. Atrial flutter and atrial fibrillation ablation - sequential or combined? A cost-benefit and risk analysis of primary prevention pulmonary vein ablation. Heart Rhythm. 2016 Jul;13(7):1441-8.
12. Kataoka N, Imamura T. How to demonstrate the advantage of cryoballoon pulmonary vein isolation over cavotricuspid isthmus ablation in patients with typical atrial flutter. J Interv Card Electrophysiol. 2024 May 30.
13. Aker A., Chernyaha-Royko U., Sorokivskyy M, Kravchuk B, Tumak I, Ivaniv Y, Zharinov O. Ablation outcomes and quality of life in patients with atrial flutter and concomitant paroxysmal atrial fibrillation. Heart, Vessels and Transplantation. 2024; 8(2):200-207

**Supplementary table 4** – Definition of outcomes in the included RCTs.

|  | **Any atrial arrhythmia** | **Typical FLA recurrence** | **New onset/ recurrent AF** |
| --- | --- | --- | --- |
| **Gupta et al. 2023** | Symptomatic atrial arrhythmia (AFL, AF, AT) > 30 sec after blanking | Symptomatic AFL | Symptomatic new onset AF |
| **Anselme et al. 2021** | *NR* | AFL > 30 sec (no blanking) | AF > 30 sec (after blanking) |
| **Mohanty et al. 2015** | Any atrial arrhythmia (AF, AFL, organized AT) > 30 sec after blanking | *NR* | *NR* |
| **Schneider et al. 2015** | Any atrial arrythmia (no blanking) | Recurrent AFL (after blanking) | *NR* |
| **Steinberg et al. 2014** | Any atrial arrhythmia with monthly burden > 0.5% after blanking | Recurrent AFL after blanking | New onset AF after blanking |
| **Mohanty et al. 2013** | Any atrial arrhythmia > 30 sec after blanking | *NR* | *NR* |
| **Navarrete et al. 2011** | Any atrial arrhythmia > 30 sec after blanking | *NR* | AF recurrence after blanking |

Abbreviations as in Table 1

**Supplementary table 5** – Quality of life assessment before and after intervention.

|  | **PVI±CTI** | **CTI** | **Mean difference (95% CI)** |
| --- | --- | --- | --- |
| **Gupta et al 2023** | | | |
| Change in EQ-5D score (mean±SD) | −0.033±0.122 | −0.030±0.122 | −0.18 (−0.91 to 0.55) |
| Change in EQ-VAS score (mean±SD) | −4.56±15.8 | −0.69±21.0 | −0.003 (−0.008 to 0.003) |
| **Mohanty et al 2013** | | | |
| Change in PF (mean)* | 3.3 | 3.9 | P-value for difference = 0.72 ** |
| Change in RP (mean)* | 15.6 | 7.9 | P-value for difference = 0.02 ** |
| Change in VT (mean)* | 10.5 | 10.3 | P-value for difference = 0.92 ** |
| Change in MH (mean)* | 3.5 | 2.8 | P-value for difference = 0.53 ** |
| Change in SF (mean)* | 8.3 | 6.5 | P-value for difference = 0.44 ** |
| Change in RE (mean)* | 13.4 | 6.1 | P-value for difference = 0.02 ** |
| Change in GH (mean)* | 4.3 | 4 | P-value for difference = 0.83 ** |
| Change in HAD anxiety (mean)* | -0.9 | -0.7 | P-value for difference = 0.25 ** |
| Change in HAD depression (mean)* | -1.2 | -0.9 | P-value for difference = 0.19 ** |
| Change in BDI score (mean)* | -1.6 | -1.0 | P-value for difference = 0.05 ** |

* Standard deviations not reported

** Mean difference and 95% CI not reported

BDI: Beck depression inventory, GH: general health, HAD: Hospital Anxiety and Depression, MH: mental health, PF: physical functioning, RE: role limitations resulting from emotional problem, RP: role limitations resulting from physical health, SF: social functioning, VT: vitality. Other abbreviations as in Table 1.

**Supplementary table 6** – Fixed-effects meta-analyses for the main outcomes.

|  | **HR** (PVI±CTI *vs*. CTI) | **95% CI** | **P-value** |
| --- | --- | --- | --- |
| **Any atrial arrhythmia recurrence** | 0.54 | 0.46-0.65 | < 0.00001 |
| **Typical AFL recurrence** | 1.68 | 0.77-3.57 | 0.19 |
| **AF recurrence/ new onset** | 0.44 | 0.32-0.61 | < 0.00001 |
| **Need for repeat ablation** | 0.53 | 0.28-0.99 | 0.05 |
| **Need for AADs during follow-up** | 0.43 | 0.35-0.53 | < 0.00001 |

Abbreviations as in Table 1

**Supplementary table 7** – Comparison of previous systematic reviews.

|  | **Gomes et al.** | **Techasatian et al.**  **JACC 2023** | **Fu et al.**  **JICE 2021** |
| --- | --- | --- | --- |
| **Type of publication** | Full text article | Congress abstract | Full text article |
| **Systematic review question** | CTI vs. PVI±CTI for typical AFL | CTI vs. PVI+CTI for typical AFL | CTI vs. PVI+CTI for typical AFL and no AF |
| **PROSPERO registration** | Yes | No | No |
| **Type of studies included** | RCT | RCT | RCT |
| **Number of studies included** | 7 | Unclear | 4 |
| **Studies’ publication date** | 2011-2023 | 2011-2018 | 2011-2018 |
| **Number of included patients** | 902 | 672 | 357 |
| **Efficacy outcomes** | Any atrial arrhythmia  Typical AFL recurrence  Incident AF  Freedom from AADs  Need for repeat ablation | Atrial arrhythmia  Freedom from AADs | Any atrial arrhythmia  Typical AFL recurrence  Incident AF  Freedom from AADs |
| **Safety outcomes** | Procedure duration  Fluoroscopy duration  Procedure-related complications | Procedure-related complications | Procedure duration  Fluoroscopy duration  Procedure-related complications |
| **Bias and quality assessment** | RoB and GRADE | None | None |
| **Conclusion** | PVI±CTI ablation is more effective than CTI alone in reducing the incidence of atrial tachyarrhythmias and subsequent AF | Prophylaxis PVI during CTI ablation is superior to CTI ablation alone in reducing atrial arrhythmias | Prophylaxis PVI during CTI ablation is superior to CTI ablation alone in reducing atrial arrhythmias |

**Supplementary figure 1** – Risk of bias assessment.


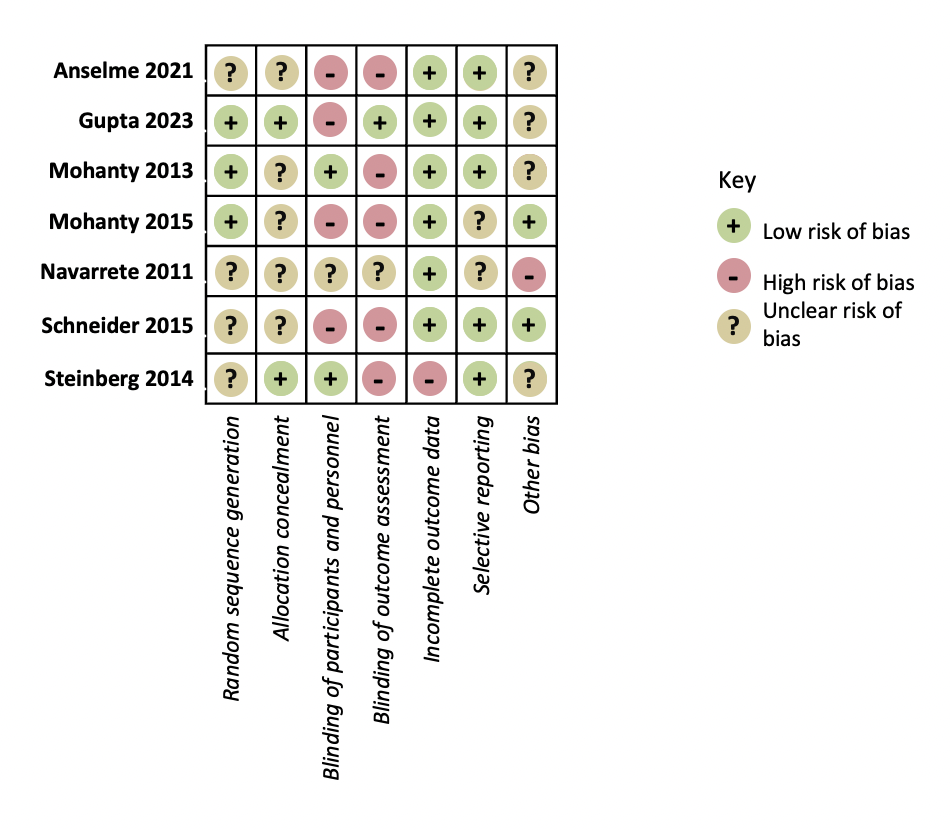


**Supplementary figure 2** – Publication bias assessment for the main outcomes: (A) recurrence of any atrial arrhythmia, (B) recurrence of typical AFL, and (C) new onset/ recurrence of AF.


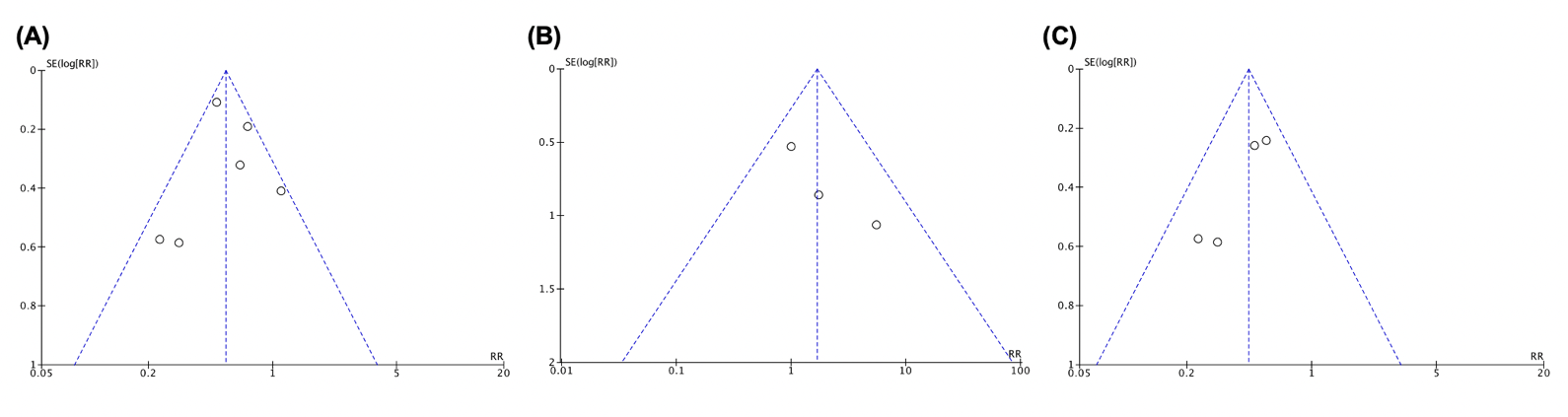


**Supplementary figure 3** – Need for repeat ablation during follow-up.


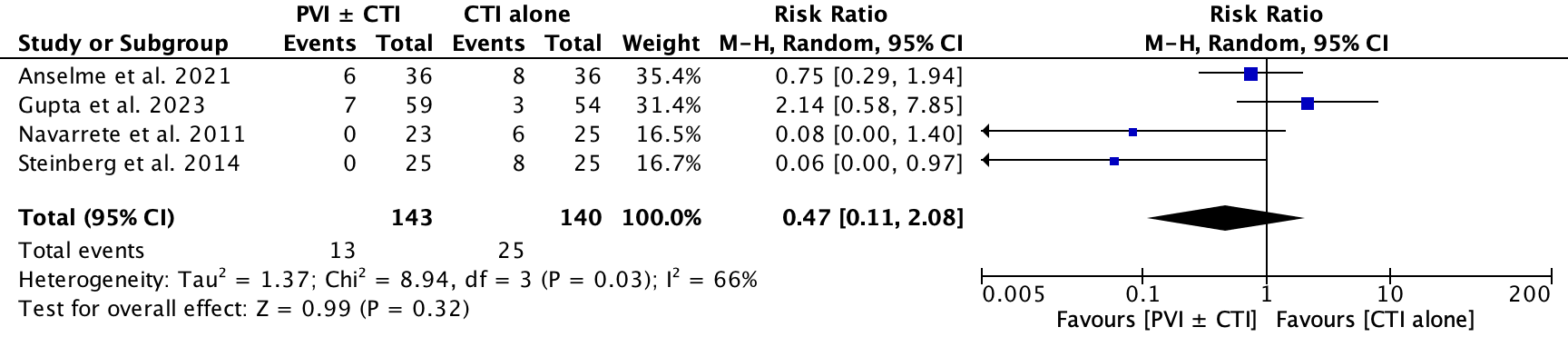


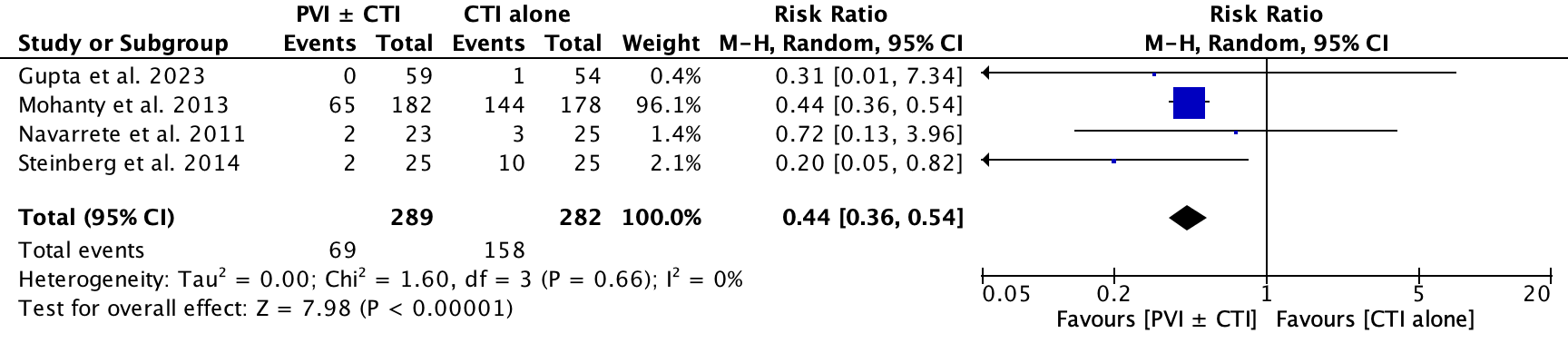
**Supplementary figure 4** – Need for AADs during follow-up.


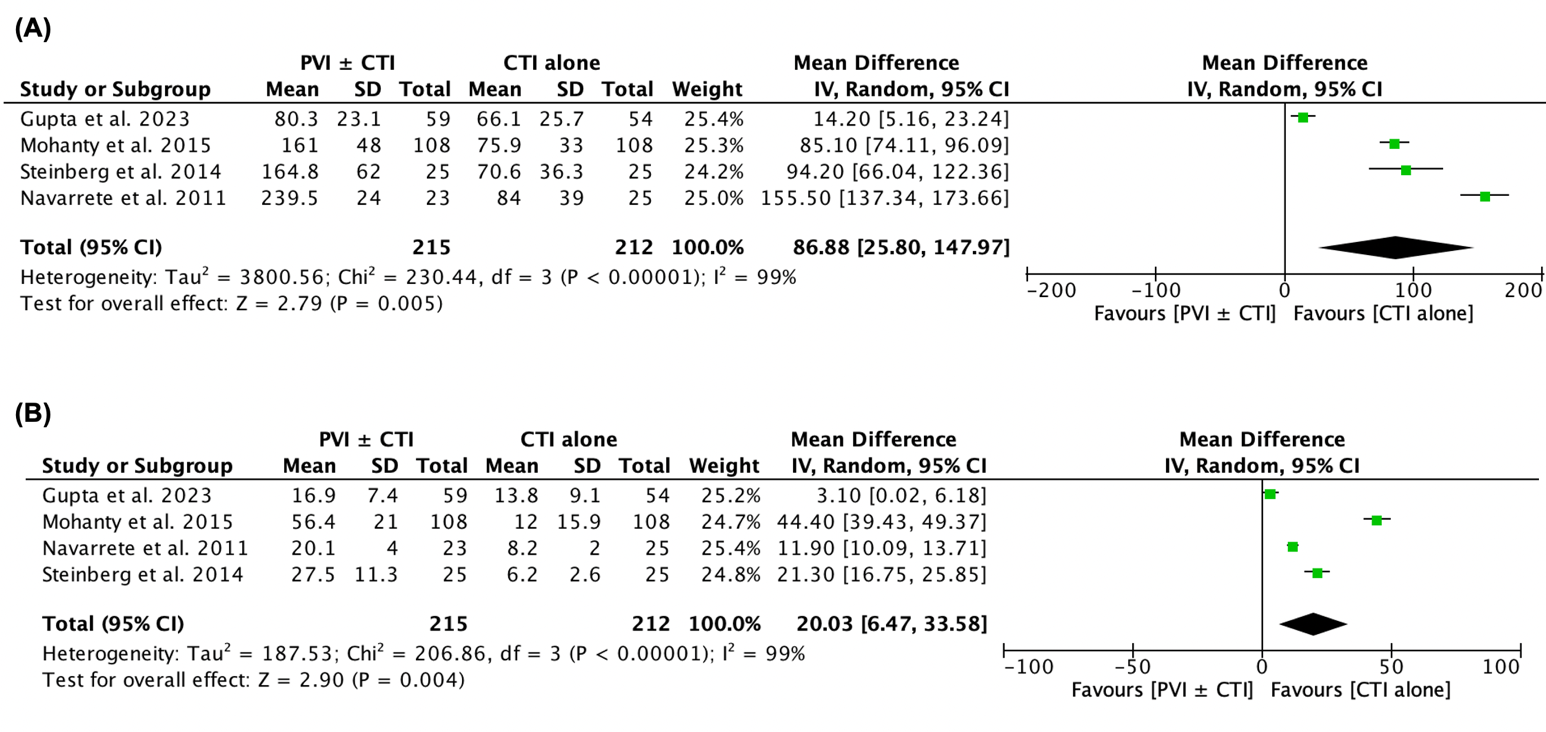
**Supplementary figure 5** – (A) Procedure and (B) fluoroscopy times (in minutes).

**
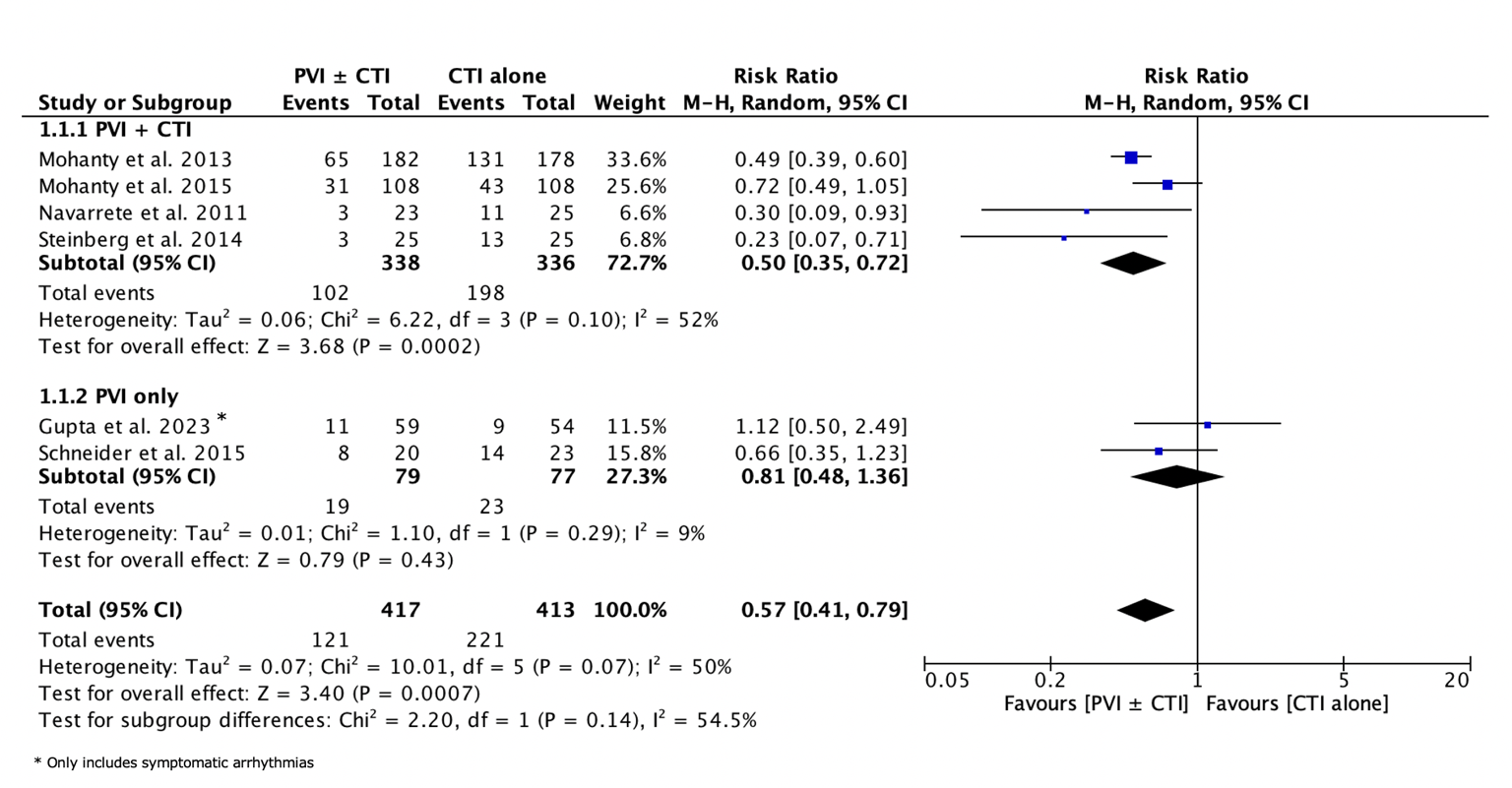
Supplementary figure 6** – Sub-group analysis for the outcome of any atrial arrhythmia recurrence according to the type of intervention (PVI + CTI or isolated PVI)


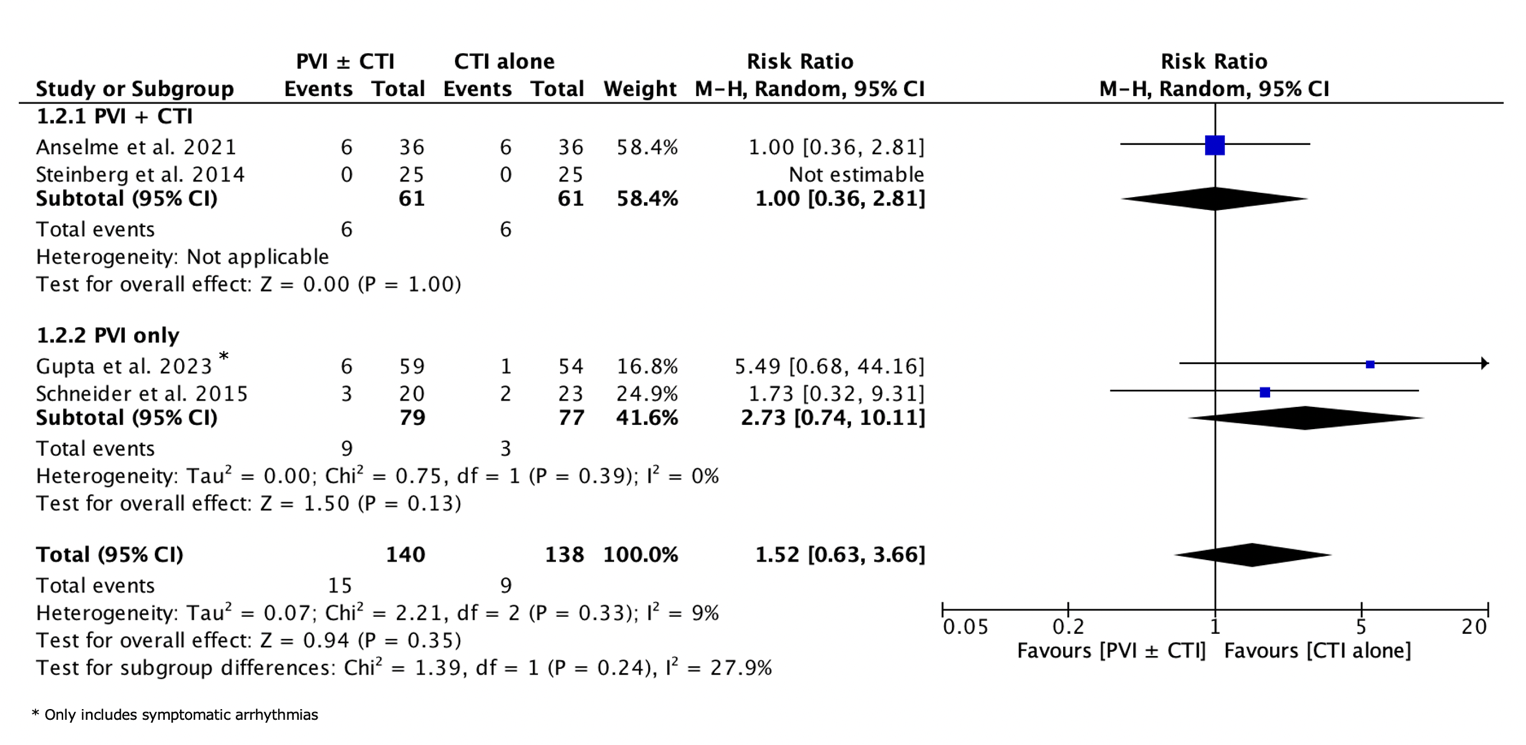
**Supplementary figure 7** – Sub-group analysis for the outcome of typical AFL recurrence according to the type of intervention (PVI + CTI or isolated PVI)


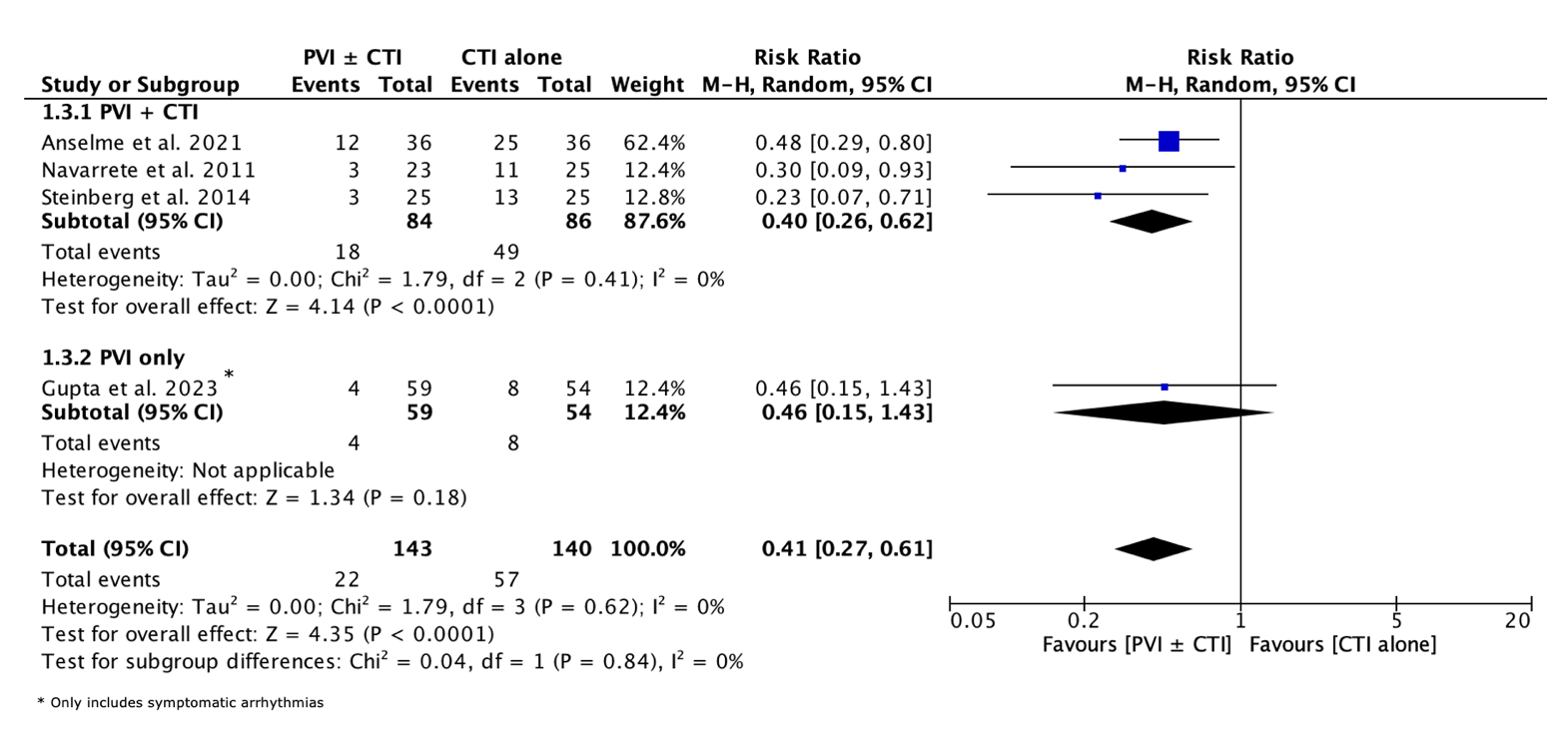
**Supplementary figure 8** – Sub-group analysis for the outcome of new onset/ recurrent AF according to the type of intervention (PVI + CTI or isolated PVI).
